# 2D Finite Element Modeling of the Cutting Force in Peripheral Milling of Cellular Metals

**DOI:** 10.3390/ma13030555

**Published:** 2020-01-23

**Authors:** Rafael Guerra Silva, Uwe Teicher, Alexander Brosius, Steffen Ihlenfeldt

**Affiliations:** 1 School of Mechanical Engineering, Pontificia Universidad Católica de Valparaíso, Av. Los Carrera, Quilpué 01567, Chile; 2 Fraunhofer Institute for Machine Tools and Forming Technology IWU Nöthnitzer Straße 44, 01187 Dresden, Germany; 3 Institute of Manufacturing Technology, Technische Universität Dresden, 01062 Dresden, Germany; alexander.brosius@tu-dresden.de; 4 Institute of Mechatronic Engineering, Technische Universität Dresden, 01062 Dresden, Germany

**Keywords:** metal foam, cellular metals, milling, finite element method

## Abstract

The machining of cellular metals has been a challenge, as the resulting surface is extremely irregular, with torn off or smeared material, poor accuracy, and subsurface damage. Although cutting experiments have been carried out on cellular materials to study the influence of cutting parameters, current analytical and experimental techniques are not suitable for the analysis of heterogeneous materials. On the other hand, the finite element (FE) method has been proven a useful resource in the analysis of heterogeneous materials, such as cellular materials, metal foams, and composites. In this study, a two-dimensional finite element model of peripheral milling for cellular metals is presented. The model considers the kinematics of peripheral milling, depicting the advance of the tool into the workpiece and the interaction between the cutting edge and the mesostructure. The model is able to simulate chip separation as well as the surface and subsurface damage on the machined surface. Although the calculated average cutting force is not accurate, the model provides a reasonable estimation of maximum cutting force. The influences of mesostructure on cutting processes are highlighted and the effects in peripheral milling of cellular materials are discussed.

## 1. Introduction

Cellular metals are a relatively young material group with a heterogeneous structure formed by a three-dimensional metallic matrix with empty pores occupying over 70% of their volume [1]. Some components could be prepared in near-net-shapes, but still some machining operations are common. 

Their field of application ranges from the chemical industry (heat exchanger, surface burner [2]) to lightweight applications with required high energy absorption capabilities [3]. Because conventional machining leads to a low surface quality and poor precision, electro-discharge machining (EDM) [4], chemical milling, or water-jet cutting are preferred for finishing [5]. Although it would be desirable to substitute them by conventional machining in order to increase productivity, it is necessary to reduce or eliminate the undesired surface defects caused by metal cutting. Previous peripheral milling tests performed for sintered cellular titanium [6] and open-cell cellular stainless steel [7] showed that machined cellular materials have highly irregular surfaces with burrs and torn off material. Additionally, these machining operations registered lower cutting forces compared to the corresponding materials that constituted their solid matrix, titanium, and stainless steel.

The measurement of surface and subsurface damage has been the subject of extensive research [8]. Attempts were done with X-ray-based computer tomographic representations of cellular structures to enable a full 3D characterization of the deformation field using digital volume correlation (DVC) to understand the impact of machining parameters on surface topography [9]. A basis for predicting the resulting surface topography consists in examining the strength of the cellular structure and the individual webs as well as their deformation behavior [10].

Some theoretical-based models have been proposed to explain the chip formation processes in cellular metals [11,12]. Nevertheless, given the limitations of the analytical and experimental methods, the finite element method has been proposed as an alternative to analyze the chip formation mechanisms and the causes of surface defects resulting from machining cellular metals [13].

Although cellular metallic materials differ fundamentally from monolithic materials with regard to their structure, the methodology and the characterization with measurements of the interrelationships on the forces acting on them are independent of the material.

In recent investigations, for example, models were developed that aim at non-linear paths under consideration of the deflection of work piece-cutter system and the continuous change of the curvature. The obtained results described the relationship that radial deflection increased with increasing cutting force and that there is a correlation to the feed rate [14]. For a non-linear path, it is also important that the engagement conditions vary considerably. A damping cutting force model was developed for this purpose. Cutting depth and the cutting speed could be identified as main parameters influencing the damping [15]. The determination of the engagement conditions, which is also very important for cellular materials, was addressed with a newly developed force model. The significance of the actual cutting geometry and the real interaction of the cutting tool and the resulting cutting forces was emphasized [16].

The special aspects of the transformation of a model from oblique cutting to peripheral milling for the prediction of cutting forces were also remarked for high-strength titanium alloys. It is important to note that the strain rate sensitivity, strain hardening, and thermal softening were considered. Despite the special characteristics of the material, predictions of the machining forces were possible, which were then verified by experimental measurements [17]. One factor that influences the machining forces in a relevant way, both experimentally and in terms of simulation, is tool wear. With the help of neural networks, it can be shown that a prognosis regarding the resulting tool loads is possible and can be applied independently of the process [18]. A prediction of the developing surface topography in relation to roughness parameters is also possible [19].

The finite element method has been used to study the chip formation of heterogeneous materials [20,21], including cellular metals [13]. Because the length scale in machining is close to the cell size, a mesoscopic model must be used for the simulation. The term mesoscopic in this context refers to models that depict the walls, webs, and cells that comprise the cellular material, which are normally in the range of 0.1 to 10 mm [22].

The object of this study is the development of a mesoscopic finite element model to explore the chip formation and damage mechanisms involved in peripheral milling. Although 2D models cannot fully reproduce the complex geometry and response of the three-dimensional mesostructure, they offer insight that cannot be obtained otherwise. Due to the high strain rates and temperatures present near the cutting edge, a constitutive model that considers these conditions is necessary. Additionally, a damage-based failure model [23] combined with the removal of failed elements was included to simulate material separation. The simulation also considers the kinematics of the process, as multiple entries of the cutting edge into the workpiece and their interaction is modeled. In order to validate the numerical model using results from milling experiments, a method based on the scale law proposed by [5] was adapted to estimate cutting forces.

In Section 2, the experimental setup and the development of the finite element (FE) model are presented. In Section 3, the results of FE simulations are shown and validated using the experimental results. In Section 4, the implications of the results are explored. Finally, in Section 5, the most important outcomes are summarized.

## 2. Materials and Methods

**Figure 1:**
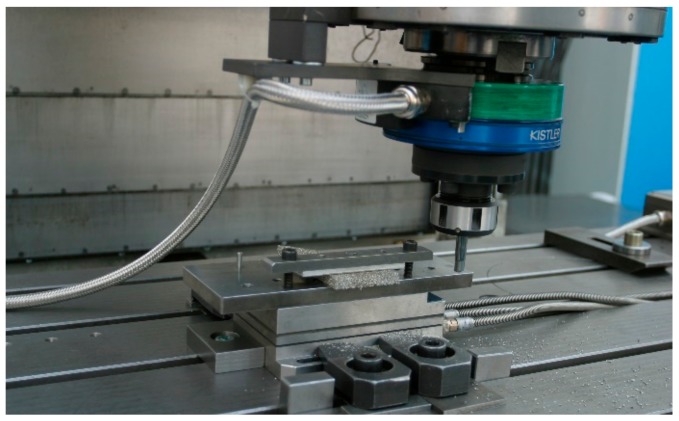
Experimental set-up for milling austenitic stainless steel cellular material (EN 1.4841, 45 ppi).

The experiments were performed with a vertical milling machine Heckert CS 800 (Werkzeugmaschinenkombinat Fritz Heckert, Karl-Marx-Stadt, GDR) with Heidenhain iTNC 530 control. Forces were measured with a Kistler rotating 4-component dynamometer 9124B (Figure 1) with a Kistler 3-component dynamometer 9257A (Kistler Instrumente AG, Winterthur, Switzerland). µCT analysis was done with a General Electric (GE) phoenix nanotom 180 (GE Sensing & Inspection Technologies GmbH, Wunstorf, Germany) with a voxel size of 4.7 µm (thickness of one slice).

**Figure 2:**
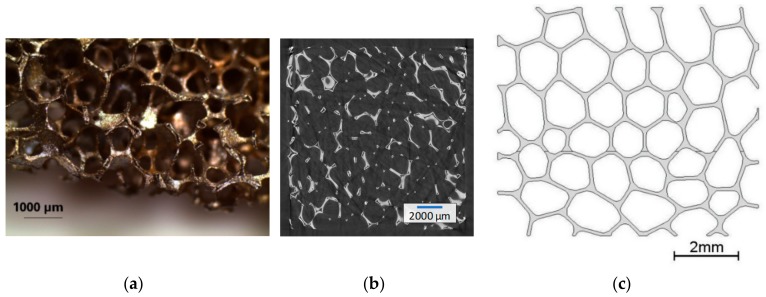
Austenitic stainless steel cellular material (**a**); one slice of a µCT-analysis of the open-cell cellular material 1.4841, 45 ppi (**b**); 2D model used in the finite element (FE) simulation, based on the stainless steel material (**c**).

An open-cell cellular material from a heat resistant austenitic stainless steel cellular material (EN 1.4841, 45 ppi, cell size 1.1–1.3 mm; relative density ρ_r_ = 0.077) was used as reference to create a FE model (Figure 2a). Its production process is based on the powder metallurgical replication technique [2].

**Figure 3:**
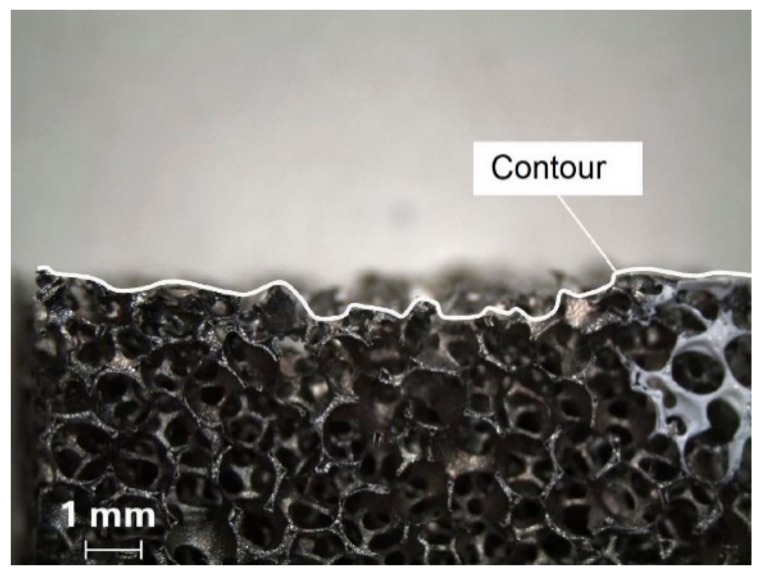
Profile of machined surface of a cellular material.

Preliminary tests were carried out to determine the extent of the fundamental surface damage (Figure 3). A mesoscopic 2D model of the cellular metal was developed (Figure 2c) based on cell form, size, strut thickness, as well as cell patterns [24] according to a microscopic analysis of the cell structure (Figure 2a) and geometrical information from µCT data (Figure 2b).

The model consists of straight struts (thickness 0.1 mm) that converge into triangle-shaped nodes. Defects such as missing struts, waviness of struts, or filled pores were not included in the model. Cutting simulations were performed on the four sides of the FE model to take into consideration the variability in measured properties in cellular metals.

**Figure 4:**
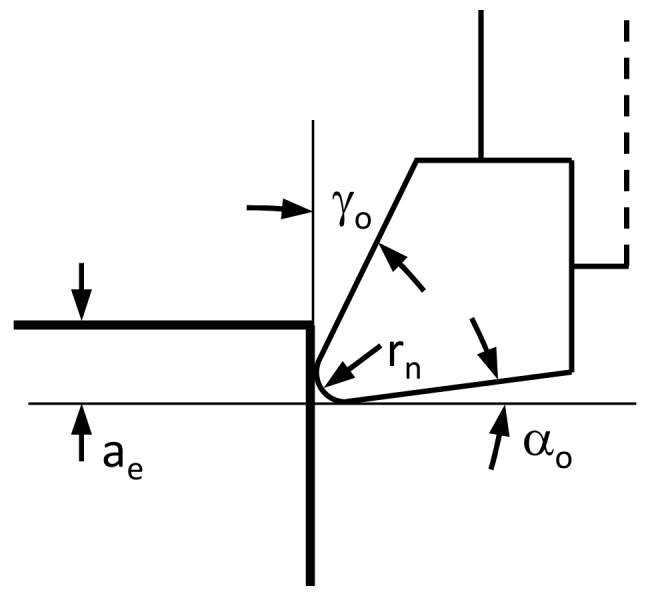
Schematic representation of the cutting edge for the 2D FE model and its parameters.

Simulations of peripheral milling were performed for both up milling and down milling. Tool geometry and cutting parameters were based on experimental data of peripheral milling tests [25]. A single-tooth milling cutter was used in the simulation (Figure 4: tool diameter D = 10 mm, cutting edge inclination angle λ_s_ = 0°, rake angle γ_o_ = 20°, relief angle α_o_ = 5°, cutting edge radius r_n_ = 10 μm). Cutting parameters used in the simulation are presented in Table 1. Cutting speed and feed were changed, while the depth of cut remained unchanged (width of cut a_e_ = 1 mm). Experimental and numerical results were compared to validate the FE model.

**Figure 5:**
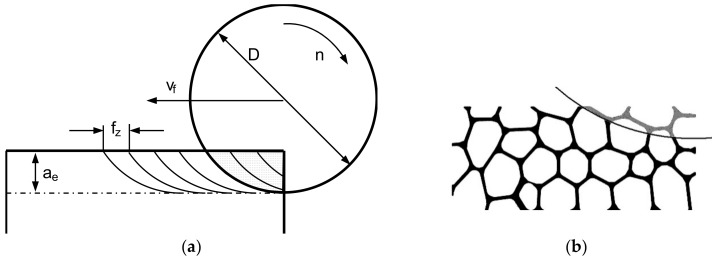
Separate tool sweeps and the material removed before the simulation (in grey) schematic representation (**a**); removed material from the FE model (in grey) (**b**).

Because the complex kinematic and geometry of the model, the operation was divided in separate tool sweeps (Figure 5a). A distance equal to the feed is set between each tooth and the following one. Each tooth is set in motion only when the previous one is no longer in contact with the workpiece or chip. Not only the cutting speed v_c_, but also the feed per tooth f_z_ were modeled. 

The numerical instability cannot be completely avoided due to the complexity of the model: as material is removed, the complex structure breaks into several fragments, each one with new surfaces. Alas, rules to define the interaction of these emerging surfaces with other bodies (i.e. chip, tool, struts) cannot be added, as the contact interactions must be prescribed before the simulation and cannot be redefined as new surfaces emerge. Hence, the simulation is limited to a maximum of five tool sweeps. Both up milling and down milling were simulated. In order to simulate material removal with a fully engaged tooth, the approach distance is not considered. The section of material in which the tool is not fully engaged is removed from the model prior to the simulation (Figure 5b).

This adjustment allows the simulation of peripheral milling at full depth of cut. On the other hand, the changes in the workpiece during the initial stage cannot be considered.

**Figure 6:**
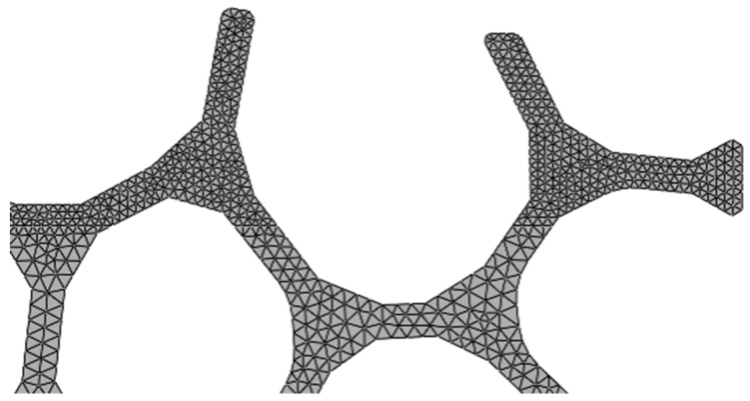
Detail of the finite element discretization for model A. A finer mesh was used in the upper region of the material, where larger strains were expected.

Four variants of the 2D model (Figure 2c) were created to accommodate the variation in the geometric structure of cellular metals, rotating and/or mirroring the reference model. Coupled temperature-displacement, 3-node linear, plane strain elements (CPE3T, Abaqus/Explicit) were used to discretized the model. Although 2D FE models cannot fully reproduce the complex three-dimensional geometry of of cellular metals, they provide insight that cannot be obtained experimentally. These models have already been used extensively to analyze the mechanical behavior of cellular materials [26,27] and also the chip formation in non-heterogeneous materials [13,20]. The number of elements and nodes used in each model are presented in Table 2. At least four elements in the cross section of struts ensure accuracy [28] in the upper region of the structure, where the larger deformations were expected (Figure 6).

The Johnson–Cook law (Equation (1)) was used to model the behavior of the basis material. The Johnson–Cook law is an empirical model often used in FE simulation of metal cutting [29,30]. The law has the form:

(1) $$\sigma =\left(A+B\varepsilon^{n}\right)\left(1+Cln\left(\dot{\varepsilon }/{\dot{\varepsilon }}_{o}\right)\right)\left(1-{\left(\left(T-T_{a}\right)/\left(T_{f}-T_{a}\right)\right)}^{m}\right),$$

where ε is the plastic deformation, $\dot{\varepsilon }$ is the deformation rate (s^−1^), ${\dot{\varepsilon }}_{o}$ is a reference rate of deformation (s^−1^), *T* is the temperature, *T_f_* is the melting temperature of the material, *T_a_* is the room temperature. Coefficient *A* is the yield stress, *B* is the hardening modulus (MPa), *C* is the coefficient of strain rate sensitivity, *n* is the hardening exponent, and *m* is the thermal softening coefficient.

The mechanical properties of a similar stainless steel, EN 1.4404, were used. The chemical composition, the mechanical and the thermophysical properties, and the Johnson–Cook coefficients for stainless steel EN 1.4404 (AISI 316L) as well as EN 1.4841 (AISI 314) are presented in Table 3, Table 4, Table 5, and Table 6.

The ductile damage model available in ABAQUS/Explicit was used to model material separation during the machining simulation [34,35,36]. The Coulomb friction model (μ = 0.2), commonly used in machining simulations, was used in the present work [37]. The penalty method was used to enforce the contact conditions at the tool–chip interface and also for the self-contact between struts. Influence of friction was not further explored.

**Figure 7:**
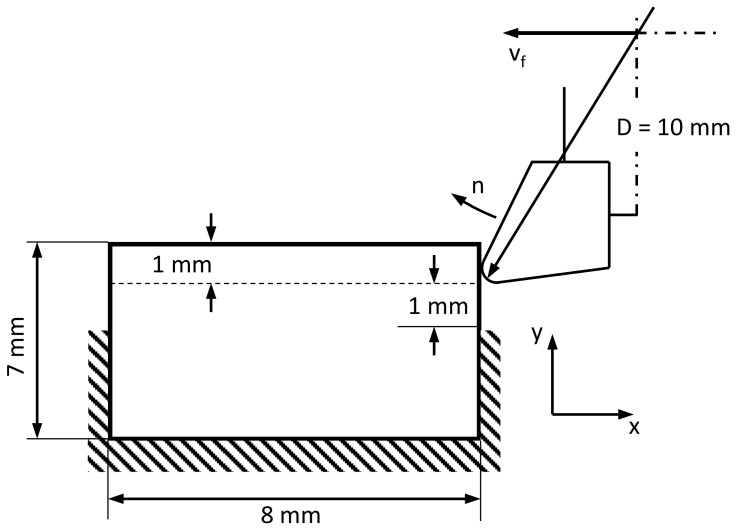
Schematic representation of the boundary conditions in the FE simulation of peripheral milling.

A 1 mm distance between fixture and tool is preserved, represented in the separation between toolpath and the fixed nodes of the lateral surfaces in the simulation (Figure 7). This preserves the deformability inside the structure while maintaining the rigidity of the structure.

The initial temperature of both workpiece and tool in the model was 25 °C. The temperature of the tool, remained constant during the simulation. The Taylor–Quinney coefficient, which defines the fraction of mechanical work transformed into heat, was set to 0.9 for the workpiece material [38,39].

## 3. Results

**Figure 8:**
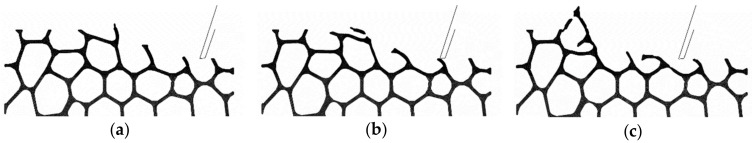
Workpiece-tool interaction (chip formation) during up milling (Model A, v_c_ = 100 m/min, f_z_ = 0.30 mm): 1st sweep (**a**); 3rd sweep (**b**); and 5th sweep (**c**).

**Figure 9:**
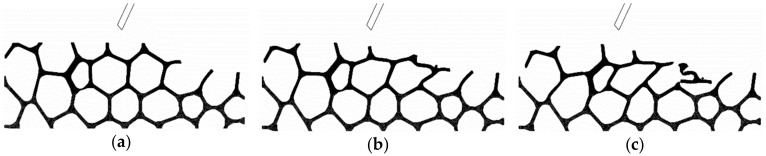
Workpiece–tool interaction (chip formation) during down milling (Model D, v_c_ = 100 m/min, f_z_ = 0.30 mm): 1st sweep (**a**); 2nd sweep (**b**); and 3rd sweep (**c**).

In Figure 8 and Figure 9, different phases of the simulation of the chip formation process in peripheral milling are presented. For both up milling and down milling, the first sweep produced no material removal, just strut deformation (Figure 8a and Figure 9a). Only after multiple sweeps some separation of material was noticeable (Figure 8c and Figure 9c).

**Figure 10:**
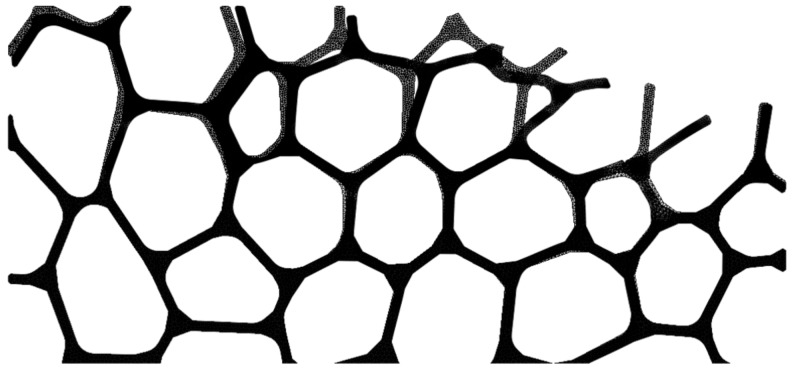
Mesostructure before (grey) and after the second sweep (black) for down milling (Model A, v_c_ = 100 m/min, f_z_ = 0.30 mm). Cell distortion is limited to cells in the upper section.

Surface damage was visible for both up and down milling; in Figure 8c, some struts were deformed, but could not be removed, and eventually filled or blocked the space of adjacent cells. Similarly, in Figure 9c the struts that were not fully removed, bent into nearby cells. In contrast to the results reported for orthogonal cutting [13], there is no noticeable evidence of subsurface damage. Deformation was limited to the cells closest to the upper surface, and distortion of struts and cells in the lower section is minimal (Figure 10).

**Figure 11:**
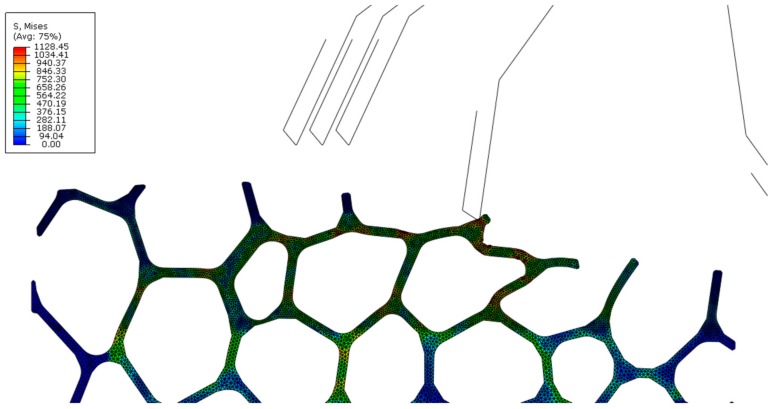
Stress distribution during down milling (von Mises in MPa, Model A, v_c_ = 100 m/min, f_z_ = 0.30 mm): during the 2nd sweep.

Figure 11 shows the distribution of von Mises stresses in cells adjacent to the surface. Stress values increased in zones near nodes and points in direct contact with the tool. Although the cutting force was distributed throughout the mesostructured, as evidenced by the stress distribution, only a limited number of struts are deformed permanently.

**Figure 12:**
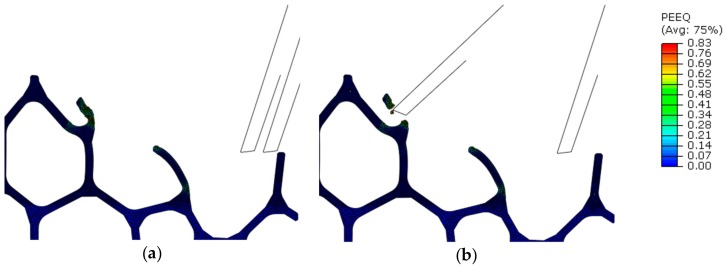
Plastic equivalent strain distribution in the region surrounding the tool path in up milling (Model A, v_c_ = 400 m/min, f_z_ = 0.03 mm): after 1st sweep (**a**) during 2nd sweep (**b**).

Figure 12 show the plastic equivalent strain distribution in the region surrounding the tool path (1st–2nd sweep). In the first sweep, the accumulated plastic strain was not enough for material separation to occur (Figure 12a), but during the second sweep plastic increased, and both strut and surface material split from the mesostructure as chip.

**Figure 13:**
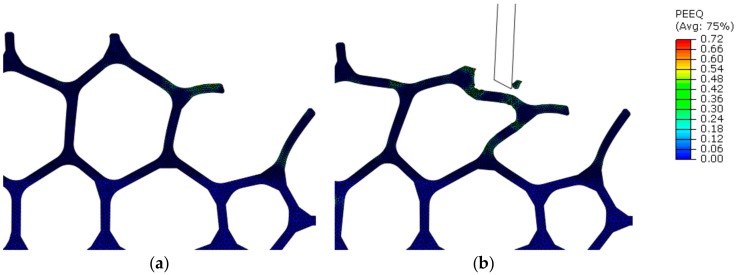
Plastic equivalent strain distribution in the region surrounding the tool path in down milling (Model A, v_c_ = 400 m/min, f_z_ = 0.03 mm): after 1st sweep (**a**) during 2nd sweep (**b**).

Plastic strain—and material separation—depended strongly on the entry point in the structure: a cantilever strut would bend (Figure 12a), while a node would offer a more rigid response and cause localized strain (Figure 12b). In down milling, node contact led to bending of adjacent cells, which also lead to an increase in accumulated plastic strain (Figure 13). In general, material separation took place only after plastic strain increased progressively during many interactions between cutting edge and strut. In some cases, plastic strain could raise rapidly in the contact zone, but only a minimal amount of material was removed.

**Figure 14:**
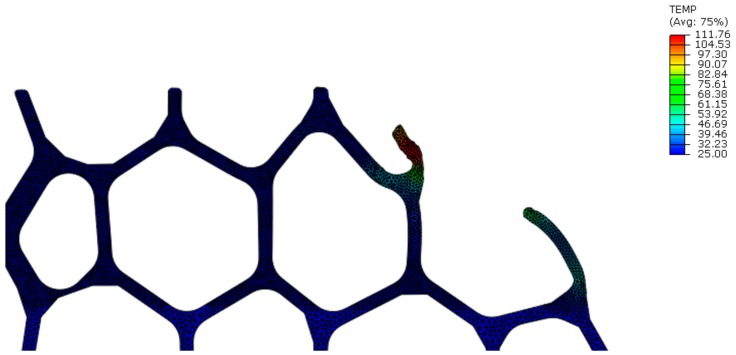
Temperature distribution in the region surrounding the tool path in up milling (temperature T (°C), Model A, v_c_ = 400 m/min, f_z_ = 0.30 mm): after 1st sweep.

Figure 14 presents the temperature distribution in the region surrounding the tool path in down milling after the first sweep. Temperature remains under 120 °C, and the increase in temperature coincides with the plastic strain distribution, as the work related to the plastic deformation turned into heat according to the assumptions imposed by the thermomechanical model. Consequently, temperature increased as the accumulated plastic strain increased in the mesostructured, reaching a maximum of 245 °C.

**Figure 15:**
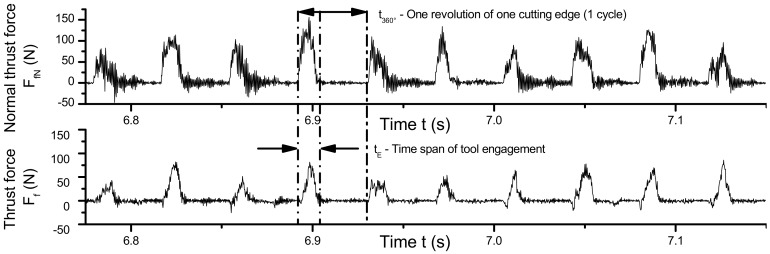
Example of the force signals measured during milling test of cellular stainless steel (1.4841, 45 ppi, f_z_ = 1 mm, a_e_ = 5 mm, v_c_ = 50 m/min).

Resulting average thrust force F_f_ and normal thrust force F_fN_ are presented in Table 7. Because in the FE simulation only the time of tool engagement was simulated and considered in the calculation of the average forces, the remaining time of the revolution was not considered, in opposition to the experimental setup in which the forces are measured uninterruptedly (Figure 15). Therefore, it is necessary to adjust the force considering a full revolution.

The angle of engagement in peripheral milling is:

(2) $$cos\varphi_{E}=1-2\cdot \frac{a_{e}}{D}.$$

When calculating the average force components, the time of one revolution t_360°_ of the tool should be considered instead of the time of engagement *t_E_*. Assuming that the reaction forces drops to zero when contact is interrupted (Figure 9), the ratio between the period of one revolution *t_360°_*, and the engagement time *t_E_*, i.e., simulation time, can be derived from the engagement angle:

(3) $$\frac{t_{E}}{t_{360{}^{\circ}}}=\frac{\varphi_{E}}{360{}^{\circ}}.$$

Consequently, the average forces can be calculated as follows:

(4) $$F_{avg}=F\cdot \frac{t_{E}}{t_{360{}^{\circ}}}.$$

Additionally, the average force components of FE simulations and machining tests were normalized dividing by the workpiece width:

(5) $$\left|F\right|=\frac{F_{avg}}{b},$$

where b = 0.05 mm for the FE simulation and b = 10 mm for the milling tests. Afterwards, the difference in relative density was considered. The relative density of the finite element model (*ρ_rFEM_* = 0.170) was greater than that of experimental samples (*ρ_rEXP_* = 0.077). 

Although the feed per tooth is smaller than the cell size, and thus the material cannot be considered as a macroscopic continuum—a basic rule of the scaling law—the force components were scaled in the analysis of the peripheral milling. This was made in order to compensate not just the different relative density, but also the shortcomings of the 2D model, which cannot replicate entirely the complexity of the three-dimensional structure.

Assuming that the scaling law adopted by [13] is also valid for milling operations:

(6) $$F_{1}\cdot \rho_{r1}{}^{\frac{3}{2}}=F_{2}\cdot \rho_{r2}{}^{\frac{3}{2}},$$

then the scaled force components can be calculated from the normalized values:

(7) ⟨F⟩=F⋅ρrEXPρrFEM32.

The normalized experimental and numerical results of feed force and normal force are presented in Table 8.

The FE-calculated values of feed and normal force differ significantly from the experimental values, overestimating the force in both cases. In contrast to the simulation of orthogonal cutting [13], the FE model cannot predict correctly the forces in peripheral milling.

Because the calculation of the reaction forces in down milling produced spurious values of cutting and thrust forces in models B, C, and D, torque (M) is used instead to calculate the cutting force:

(8) $$F=\frac{M}{D}.$$

Table 9 shows the average cutting force according to Equation (8) for up milling and down milling derived from the values of torque. The cutting force is normalized considering both workpiece width (Equation (5)) and relative density (Equation (7)).

## 4. Discussion

One of the many challenges in the development of an FE model for the analysis of large deformations of cellular metals is the simulation of contact interactions between inner surfaces. This issue is worsened as fracture sets in and new surfaces are revealed. As new contact pairs emerge, the model becomes a multibody problem, which includes contact mechanics, plasticity, and material separation.

Since a finite element simulation of the entire peripheral milling was not possible, an analysis of the surface quality using the FE model was not possible either. Nevertheless, some insights into the chip formation process are possible. Plastic strain—and material separation—depended strongly on the entry point: more rigid regions will lead to higher strains and eventually to material separation, while less rigid regions will bend or twist. Material separation could only occur after plastic strain increased progressively as the cutting edge progressed along the surface. Chip was constituted mostly of small fragments of struts. This could be a consequence of the relatively small feed, as tool engagement is limited and the effect on nearby cells is damped.

Surface damage was visible in all cases, as struts were bent and displaced into adjacent cells. However, there was no clear evidence of subsurface damage. Deformation was limited to the cells closest to the upper surface, and distortion of struts and cells in the lower section was minimal. There were also signs of elastic recovery in slender struts, which could alter the resulting machined surface/contour. Nevertheless, due to the limited time lapse of the simulation, it is hard to ascertain whether cutting parameters might influence the resulting surface quality via FE models. 

According to the FE simulation, temperature remained relatively low (under 250 °C) when compared to the machining of solid stainless steel. As there were no clearly defined primary or secondary deformation zones, and interaction between workpiece and tool were negligible, the effect of friction on temperature should be limited. Hence, any increase in temperature depends exclusively on the heat generated by the plastic strain. As plastic strain is localized, it is reasonable to expect lower temperatures. Nevertheless, as temperature measurements were not carried out during the machining experiments, the validity of numerical results must be confirmed.

The cutting forces were not accurately calculated by the model, and the values are over four times higher for down milling when compared to experimental results. The deviation was even larger for up milling.

Other deviation between the FE model and the experimental results can be appreciated in Table 8. The model reflects an increase in cutting force for higher values of cutting speed and feed, in opposition to the experimental results, in which no relation between cutting speed and cutting force could be established.

The deviation could be traced back to three factors. First, density and configuration of both structures are different. While the scaling law was adequate in orthogonal cutting, the differences between real structure and model become more relevant due to the chip thickness to cell size ratio (1:3 in milling vs 1:1 in orthogonal cutting). Second, the highly idealized mesostructure of the model, which does not take into consideration defects such as missing or irregular struts, which are extremely common in cellular metals. Third, the limited knowledge about the basis material at high strain rates, as the sintered material in the struts should have inferior mechanical properties than those reported by [31,33]. Additionally, the insufficient knowledge of the applicable material laws and effective contact conditions between tool and material poses a challenge. It is known, for example, that knowledge about the friction between the contact partners drastically influences the simulation results [37].

## 5. Conclusions

A 2D mesoscopic FE model to analyze the peripheral milling of cellular metals is presented. The model included the kinematics of peripheral milling, depicting the movement of the tool—into the workpiece and contact interaction between the cutting edge and the mesostructure. The model was capable of simulating chip separation as well as portray the subsurface damage mechanisms that take place in peripheral milling.

The model allows only a limited analysis of the mechanisms that lead to surface defects such as burrs and tearing off of material, as the simulation is heavily constrained by the complex geometry and the non-linear thermomechanical response of the material.

Although the calculated average cutting force was not accurate, the FE model provided a reasonable estimation of maximum cutting force. The influence of mesostructure on the chip formation process is crucial, as cutting force differed strongly depending on the arrangement of cells. The role of the mesostructure might pose a challenge in the quest for better surface quality and precision in the machined surfaces of cellular metals, as the cell arrangement defines the functionality of the material and cannot be changed at will.

As there are no available analytical or empirical models for the machining of cellular metals, FE models could be a useful resource for the design and optimization of such processes. Nevertheless, improvements are required, as deviations could be traced back to the assumptions of the numerical model: two-dimensional instead of three-dimensional, lack of geometrical defects, and the uncertainty regarding the mechanical properties of the sintered material constituting the struts.

## Figures and Tables

**Table 1:** Cutting parameters in the FE simulations of peripheral milling.

Feed Per Tooth f_z_ (mm)	Cutting Speed v_c_ (m/min)
0.05	100
0.05	400
0.30	100
0.30	400

**Table 2:** Number of elements and nodes in the different 2D FE models of milling.

Model	Elements	Nodes
A	12 376	7 819
B	14 969	9 318
C	16 624	10 255
D	15 810	9 867

**Table 3:** Chemical composition of the steel substrates according to EN10088-1.

	Chemical Composition (Mass %)					
Steel Name (Steel Number)	C	Si ≤	Mn ≤	Cr	Ni	Mo
X2CrNiMo17-12-2 (1.4404)	0.03	1.00	2.00	16.5–18.5	10.0–13.0	2.00–2.50
X15CrNiSi25-21 (1.4841)	0.45	1.5–2.5	2.00	24–26	19–22	—

**Table 4:** Thermo-physical properties of the stainless steel 316L [31].

Property	Formula
Density ρ (kg/m^3^)	ρ (T) = 7921 – 0.614 × T + 0.0002 × T^2^
Thermal conductivity λ (W/mK)	λ (T)= 14.307 + 0.0181 × T – 6 × 10^-6^ × T^2^
Specific heat c_p_ (J/kg K)	c_p_ (T) = 440.79 + 0.5807 × T – 0.001 × T^2^ + 7 × 10^-7^ × T^3^

**Table 5:** Temperature-dependent Young’s modulus and Poisson’s ratio of the sintered stainless steel 316L [32].

Temperature T (°C)	20	100	200	400	600
Young’s modulus E (GPa)	193	192	185	168	151
Poisson’s ratio ν	0.27	0.27	0.27	0.27	0.27

**Table 6:** AISI 316L material constants for J–C constitutive model [33].

A	B	C	N	m	e_0_
514	514	0.042	0.508	0.533	0.001

**Table 7:** Average reaction forces for the peripheral milling (Model A, v_c_ = 100 m/min, f_z_ = 0.05 mm).

	Down Milling		Up Milling	
Sweep	F_f_ (N)	F_fN_ (N)	F_f_ (N)	F_fN_ (N)
1	0.07	−0.05	−0.15	0.11
2	0.04	−0.03	−0.06	0.04
3	0.04	−0.04	−0.06	0.04
4	0.05	−0.03	−0.06	0.03
5	0.07	−0.04	−0.22	0.20
Average	0.054	−0.038	−0.110	0.084

**Table 8:** Scaled average reaction forces for the peripheral milling (Model A, v_c_ = 100 m/min, f_z_ = 0.05 mm).

	Down Milling		Up Milling	
	⟨F_f_⟩ (Nmm^−1^)	⟨F_fN_⟩ (Nmm^−1^)	⟨F_f_⟩ (Nmm^−1^)	⟨F_fN_⟩ (Nmm^−1^)
Experimental	0.077	0.058	0.018	0.066
Simulation	0.349	0.242	0.675	0.529

**Table 9:** Influence of cutting parameters on cutting force in peripheral milling (FE model).

Cutting Speed v_c_ (m/min)	Feed Per Tooth f_z_ (mm)	Cutting Force F_c_ (N)	
		Down Milling	Up Milling
100	0.05	1.204	0.823
100	0.30	2.119	1.549
400	0.30	2.503	2.716

## Nomenclature

a_e_	mm	Width of cut
ρ_r_	—	Relative density
D	mm	Tool diameter
λ_s_	°	Cutting edge inclination angle (helix angle)
γ_o_	°	(Orthogonal) rake angle
α_o_	°	Relief angle
r_n_	µm	Cutting edge radius
f_z_	mm	Feed per tooth
v_c_	m/min	Cutting speed
n	1/min	Rotational speed
v_f_	mm/min	Feed rate
ε	—	Plastic deformation
$\dot{\varepsilon }$	1/s	Strain rate
${\dot{\varepsilon }}_{o}$	1/s	Reference strain rate
T	°C	Temperature
T_f_	°C	Melting temperature
T_a_	°C	Room temperature
A	MPa	Yield stress
B	MPa	Hardening modulus
C	—	Strain rate sensitivity
n	—	Hardening exponent
m	—	Thermal softening exponent
ρ	kg/m³	Mass density
λ	W/m∙K	Thermal conductivity
c_p_	J/kg∙K	Specific heat
E	GPa	Young’s modulus
ν	—	Poisson ratio
F_f_	N	Thrust force
F_fN_	N	Normal thrust force
F_c_	N	Cutting force
ϕ_E_	°	Arc of engagement
t_E_	s	Time
t_360°_	s	Engagement time of one revolution (360°)
F_avg_	N	Average force
b	mm	Width of cutting engagement (similar to a_p_)
$\left|F\right|$	N/mm	Width scaled force
⟨F⟩	N/mm	Width and relative density scaled force
M	Nm	Torque
µ	—	Friction coefficient
